# Phase I dose-escalation study of pazopanib combined with bevacizumab in patients with metastatic renal cell carcinoma or other advanced tumors

**DOI:** 10.1186/s12885-017-3527-7

**Published:** 2017-08-15

**Authors:** Sylvie Négrier, David Pérol, Rastislav Bahleda, Antoine Hollebecque, Etienne Chatelut, Helen Boyle, Philippe Cassier, Séverine Metzger, Ellen Blanc, Jean-Charles Soria, Bernard Escudier

**Affiliations:** 1 University Lyon, Université Claude Bernard Lyon 1, Centre Léon Bérard, Lyon, France; 2 Clinical Research and Innovation Department, Centre Léon Bérard, F-69373, Lyon, Cedex 08 France; 3 DITEP -Département d’Innovation Thérapeutiques et Essais Précoces, Institut Gustave Roussy, 94805 Villejuif Cedex, France; 4 Institut Claudius Regaud, Inserm UMR1037 CRCT, Université Paul-Sabatier, 20/24 rue du Pont Saint-Pierre, 31052 Toulouse, France; 5 Medical Oncology Department, Centre Léon Bérard, F-69373, Lyon, Cedex 08 France; 6 University of Paris Sud, Orsay, Institut Gustave Roussy, 94805 Villejuif Cedex, France; 7 Department of Medical Oncology, Institut Gustave Roussy, 114, rue Edouard-Vaillant, 94805 Villejuif Cedex, France

**Keywords:** Renal carcinoma, Bevacizumab, Pazopanib, Combination Angiogenesis, Phase I trial

## Abstract

**Background:**

Vascular endothelial growth factor (VEGF) directed therapies are being used in a large number of advanced tumors. Metastatic renal cell carcinoma (mRCC) is highly dependent on the VEGF pathway; VEGF receptor (VEGFR) tyrosine kinase inhibitors (TKI) and humanized VEGF monoclonal antibody have been registered for clinical use in advanced renal cell carcinoma. The VEGFR TKI, pazopanib, with a rather manageable toxicity profile, was preferred to sunitinib by mRCC patients. We investigate the combination of pazopanib and bevacizumab to determine the maximum tolerated dose (MTD) in mRCC and other advanced solid tumors.

**Methods:**

In this bicentric phase I trial with a 3 + 3 + 3 dose-escalation design, patients received oral pazopanib once daily plus intravenous infusion of bevacizumab every 2 weeks from D15, at one of the four dose levels (DL) planned according to the occurrence of dose limiting toxicities (DLT). 400 and 600 mg pazopanib were respectively combined with 7.5 mg/kg bevacizumab in DL1 and DL2, and 600 and 800 mg pazopanib with 10 mg/kg bevacizumab in DL3 and DL4. Tumor response was evaluated every 8 weeks. Blood samples were assayed to investigate pazopanib pharmacokinetics.

**Results:**

Twenty five patients including seven mRCC were enrolled. Nine patients received the DL1, ten received the DL2. No DLT were observed at DL1, five DLT at DL2, and 3 DLT in the six additional patients who received the DL1. A grade 3 microangiopathic hemolytic anemia syndrome was observed in four (16%) patients. Five (22%) patients achieved a partial response. The mean (range) plasmatic concentrations of 400 and 600 pazopanib were respectively 283 (139–427) and 494 (227–761) μg.h/mL at Day 1, and 738 (487–989) and 1071 (678–1464) μg.h/mL at Day 15 i.e. higher than those previously reported with pazopanib, and were not directly influenced by bevacizumab infusion.

**Conclusions:**

The combination of pazopanib and bevacizumab induces angiogenic toxicity in patients without any pre-existing renal or vascular damage. Even if a marginal efficacy was reported with five (22%) patients in partial response in different tumor types, the toxicity profile compromises the development of this combination.

**Trial registration:**

The study was retrospectively registered on ClinicalTrials.gov (number NCT01202032) on 2010, Sept 14th.

## Background

The efficacy of an anti-VEGF antibody was originally demonstrated in renal cell carcinoma and published forteen years ago [1]. Treatments have evolved from known therapies using exclusively cytokines to therapies targeting angiogenesis, cell proliferation, and tumor growth. These recent developments have enabled tangible clinical benefits in different solid tumor types [2–5], especially in renal cell cancer, and supported subsequent development of VEGF inhibitors, mainly tyrosine kinase inhibitors (TKI) directed against VEGF receptors (VEGFR). Different agents targeting the VEGF pathway are currently registered for the treatment of advanced renal cell cancer patients [6–13]. Despite improvements observed with these targeted treatments especially in progression free survival duration, the tumor sensitivity to drugs remains limited with only scarce complete responses observed and over time resistance arises. The combination of different agents has emerged as an interesting strategy to potentially enhance the efficiency of the treatments and delay the disease’s progression due to drug resistance. Combinations of VEGF inhibitors and mTor inhibitors or cytokines, administrated to patients with renal cell cancer, were acceptable in terms of tolerance but no additional gain was achieved [14–19] until recently. Indeed, the combination of lenvatinib and everolimus recently re-opened the hypothesis of a synergic combination of VEGFR and mTor inhibitors for the treatment of mRCC [13, 20]. The combination of VEGFR TKI with a VEGF-directed antibody also looks promising but increases the treatment-related toxicity. A rather strong rational supports the combination of bevacizumab known to induce a rapid clearance of circulating VEGF, with VEGFR TKIs that mostly induce an increase of the circulating VEGF levels. High serum or plasmatic levels of VEGF were indeed previously correlated with tumor progression [18, 21–23]. The potential binding of VEGF to other receptors such as the platelet-derived-growth-factor receptor (PDGFR) might also contribute to the virtually constant acquired resistance in patients treated with a VEGFR inhibitor [24]. The concomitant blockade of VEGF ligand and receptors might contribute to improve the treatment efficacy. Some of these combinations have been attempted and reported promising results in terms of efficacy but their feasibility remains as a matter of debate [14, 25–28].

Pazopanib, one of the most recently registered TKI for first-line advanced renal cancer treatment, is known to target VEGFR-1, −2, and −3, PDGFR-α and –β as well as c-KIT [29]. Its safety profile slightly differs from that of the commonly used sunitinib. With a better tolerance reported with this multitargeted TKI, pazopanib appeared as a promising candidate to be used in combination with bevacizumab. This latter intravenous agent was also registered for treatment in metastatic renal cell cancer (mRCC) patients in combination with interferon α [7, 30]. Some activity was also demonstrated when used as monotherapy in these patients [31, 32]. Discordant results in terms of efficacy were previously reported with the combination of sunitinib and bevacizumab according to the tumor type [25, 26, 33, 34]. This phase I combination trial was consequently not only conducted in renal cell cancer patients but also in patients with other tumor types. The aim of the PARASOL trial was to test the feasibility of the combination of pazopanib with bevacizumab and to investigate pazopanib pharmacokinetics (PK).

## Methods

### Patients

Adult patients with histologically confirmed diagnosis of solid tumor excluding squamous non-small-cell lung cancer because of an increased bleeding risk [33, 34], and refractory to a maximum of two lines of standard treatments, or without prior treatment for renal cell carcinoma were eligible. Additional inclusion criteria were Eastern Cooperative Oncology Group performance status (ECOG-PS) of 0 or 1, adequate vital functions defined as absolute neutrophil count ≥1500 cells/μL, hemoglobin ≥9.0 g/dL, and platelets ≥100,000 cells/μL, PT ≤1.2xupper limit of normal [ULN] and APTT ≤1.2xULN, hepatic aspartate aminotransferase (AST) / alanine aminotransferase (ALT) ≤2.5xULN, total bilirubin ≤1.5xULN, and serum creatinine ≤1.5 mg/dL or creatinine clearance ≥50 mL/min. Patients with insufficiently controlled blood pressure, increased proteinuria (>1.0 g/L), history of acute cardiac event, coronary disease or stroke in the previous 6 months, or corrected QT (QTc) interval prolongation (>480 ms using Bazett’s formula), and patients with history of brain metastases were excluded.

The study was conducted according to the declaration of Helsinki and the International Conference of Good Clinical Practices after local approval of the Ethic Committee of Lyon Sud-Est IV and all patients provided written informed consent before enrollment. The study was registered on ClinicalTrials.gov, number NCT01202032.

### Study design

**Fig. 1:**
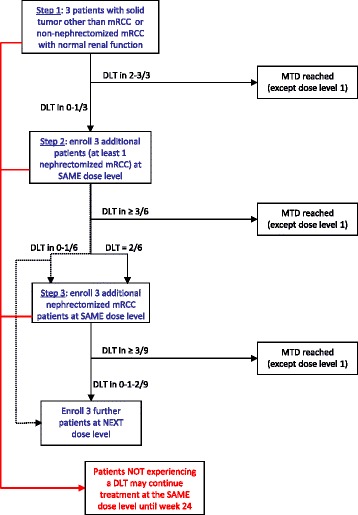
Study schedule (3 + 3 + 3 escalation steps)

This investigator-initiated phase I trial with a 3 + 3 + 3 dose-escalation design was conducted in two institutions. Cohorts of three to nine patients were sequentially enrolled to receive one of the three escalated doses of pazopanib combined with two escalated doses of bevacizumab. The main objective was to determine the maximum-tolerated dose (MTD) of the combination in patients with advanced renal cell carcinoma or with other advanced tumors. MTD was defined as the highest dose level (DL) at which less than two of nine patients experienced a dose-limiting toxicity (DLT) during the first 8 weeks. Secondary objectives were the objective response rate based on RECIST 1.1 criteria [35], the 6-month progression-free survival rate, and the pharmacokinetics (PK) of pazopanib in this combination. Cohorts of patients were enrolled in three successive steps according to the study plan shown on Fig. 1. Enrollment of nephrectomized mRCC patients were forbidden in the first step but allowed in the following steps (at least one patient in the second, and three patients in the third step). According to the independent Data and Safety Monitoring Committee (DSMB), patients enrolled at the third step of DL 2 and beyond must not have been nephrectomized. Patients were allowed to pursue the experimental treatment until tumor progression as long as the tolerance was acceptable. Safety analyses were performed after the 19th inclusion, and the steering committee, in agreement with the DSMB, recommended an extension cohort of six non-nephrectomized patients to be treated at the first dose level (400 mg pazopanib, bevacizumab 7.5 mg/kg) in order to confirm the MTD.

### Treatment and dose escalation plan

Patients received oral pazopanib (Votrient®) (Novartis, Rueil-Malmaison, France) once daily at a dose of 400, 600 or 800 mg per day according to the dose level plan, and intravenous bevacizumab (Avastin®) (Roche, Boulogne-Billancourt, France) at 7.5 or 10 mg/kg every 2 weeks (Q2W). Bevacizumab injections started 2 weeks after pazopanib initiation. Toxicity was assessed according to the National Cancer Institute Common Terminology Criteria for Adverse Events (CTCAE) version 3.0. Escalation to the next dose cohort was allowed following safety assessment after at least the first 8 weeks and validation by the DSMB. A DLT was considered in case of any grade 4 adverse event (AE), thrombotic AE, grade 3 cardiac failure, non-controlled hypertension, thrombocytopenia, AST, ALT, or bilirubin level increase, or any other grade 3 AE lasting more than 7 days except fatigue. A 200 mg/day dose reduction of pazopanib was decided for patients experiencing a non-DLT grade 3 AE. No bevacizumab dose modification was allowed but the infusion could be delayed once. Patients requiring larger pazopanib dose reductions, or more than 4 weeks bevacizumab discontinuation were withdrawn from the study. Intra-patient dose escalation was not allowed.

### Tumor assessment

Responses were assessed according to RECIST version 1.1 [35] every 8 weeks up to 24 weeks, and every 3 months thereafter. Progression free survival (PFS) was measured from the first day of pazopanib administration until the date of progression, death, or treatment discontinuation for toxicity whichever occurred first.

### Pharmacokinetic assessments

Plasmatic concentration of pazopanib was centrally assessed on blood samples collected at different time points on day 1, before treatment, at 0.5, 1, 2, 3, 4, 6, 8, 12, and 24 h after the first pazopanib administration, and on day 14, prior to bevacizumab infusion. One serum sample was collected at week 5 and week 7 prior to bevacizumab infusions. Pharmacokinetic analyses (PK) including pazopanib area under the concentration-time curve (AUC) according to dose level, and coefficient of variation (CV%) were performed in the Pharmacology Unit of the Institut Claudius Regaud, Toulouse, France, as previously reported [36].

### Statistics and data analysis

This 3 + 3 + 3 dose-escalation study was designed to screen patients for major toxicity in a large proportion of the pazopanib and bevacizumab patient-treated population. Based on binomial probabilities, in three patients (six, and nine patients respectively) cohort, the probability to observe one or more DLT, if that DLT occurred in at least 54% (32 and 23% respectively) of the population, was 90%. A descriptive analysis was performed to describe patient demographics and clinical characteristics, occurrence of adverse events (AE), incidence per CTCAE grade and dose level, and response rates. PFS was defined as the time from the date of first study drug administration until the date of first documented progression or death from any cause, and analyzed using the Kaplan-Meier method. Censoring was applied in the following situations: lost to follow-up and no event before cut-off (Oct 7th, 2013). Associations between dose, PK variables, and toxicity were established using Pearson correlation coefficients and compared using the two-tailed t test. SAS version 9.3 was used for all statistical analyses.

## Results

### Patients

Between July 2010 and August 2012, 25 patients were enrolled including the six patients of the additional confirmatory cohort. Seven patients had mRCC (only one has not previously undergone a nephrectomy) whereas other patients had melanoma (*n* = 4), pancreatic cancer (*n* = 2), head and neck (*n* = 2) and cervix cancer (*n* = 2) (Table 1). The median (range) age was 62 (41–79) and 14 (56%) patients were males. Nine (37.5%) patients had a history of hypertension but the blood pressure was adequately controlled at the time of inclusion. The median number (range) of previous treatments in patients with other tumors than mRCC was 3 (1–6). No patient had previously received VEGFR tyrosine kinase inhibitors. One patient with metastatic breast cancer had previously received bevacizumab in combination with paclitaxel.

**Table 1:** Patient Demographics and Clinical Characteristics. Data are median (range) or n (%) unless otherwise indicated

Characteristics	Renal cell carcinoma *N* = 7 (28.0%)		Other tumor types *N* = 18 (72.0%)		Patients *N* = 25	
Median Age, years (min-max)	53.10 (43.80–71.20)		62.55 (41.00–78.60)		61.90 (41.00–78.60)	
Male	5	(71.4)	9	(50.0)	14	(56.0)
History of hypertension	3	(42.9)	6	(33.3)	9	(36.0)
Nephrectomy	6	(85.7)	1	(5.0)	7	(87.5)
Localization						
Cervix cancer	0	-	2	(11.1)	2	(8.1)
Colorectal cancer	0	-	1	(5.6)	1	(5.6)
Melanoma	0	-	4	(22.2)	4	(16.0)
Pancreatic cancer	0	-	2	(11.1)	2	(8.1)
Breast cancer	0	-	1	(5.6)	1	(5.6)
Adrenocortical carcinoma	0	-	1	(5.6)	1	(5.6)
Seminoma	0	-	1	(5.6)	1	(5.6)
Lung cancer	0	-	1	(5.6)	1	(5.6)
Other (head and neck, thyroid, bladder, mesothelioma)	0	-	5	(27.8)	5	(25.0)
M0 at diagnosis	5	(71.4)	13	(72.2)	18	(72.0)
Time between diagnosis and first metastases						
Mean (std)	11.86 (15–27)		21.73 (23–56)		18.85 (21–64)	
Median (min-max)	12.88 (−1.28–43.20)		16.29 (−0.39–80.06)		13.14 (−1.28–80.06)	
Missing	0		1		1	
Time between diagnosis and first metastases						
≤ 12 months	3	(42.9)	7	(41.2)	10	(41.7)
> 12 months	4	(57.1)	10	(58.8)	14	(58.3)
Missing	0		1		1	
Number of metastatic sites						
1	5	(71.4)	12	(70.6)	17	(70.8)
> 1	2	(28.6)	5	(29.4)	7	(29.2)
Missing	0		1		1	
Median (min-max)	1 (1–1)		3 (1–6)		3 (1–6)	
Number of prior therapy						
Chemotherapy	1	(14.3)	18	(100.0)	19	(76.0)
Median number of previous chemotherapy (min-max)	1 (1–1)		3 (1–6)		3 (1–6)	
Bevacizumab	0	(0.0)	1	(5.6)	1	(5.3)
Radiotherapy	3	(42.9)	13	(72.2)	16	(64.0)
Hemoglobin						
< 115 g/L (F), <130 g/L (M)	1	(14.3)	7	(38.9)	8	(32.0)
≥ 115 g/L (F), ≥130 g/L (M)	6	(85.7)	11	(61.1)	17	(68.0)
Serum creatinine (μmol/L)						
Median (min-max)	97.5 (84.0–127.0)		79.5 (49.0–127.0)		84.0 (49.0–127.0)	
Missing	1		0		1	
AST (UI/L)						
Median (min-max)	17.0 (16.0–21.0)		33.0 (13.0–84.0)		24.0 (13.0–84.0)	
Missing	1		0		1	
ALT (UI/L)						
Median (min-max)	21.0 (10.0–34.0)		28.0 (8.0–52.0)		26.0 (8.0–52.0)	
Missing	1		0		1	
LDH > normal	2	(28.6)	10	(55.6)	12	(48.0)

### Treatment administration

Nine patients were enrolled in the initial cohort and received 400 mg pazopanib combined with 7.5 mg/kg bevacizumab (DL1), ten patients of the second cohort received 600 mg pazopanib with 7.5 mg/kg bevacizumab (DL2) (Fig. 1). One patient was withdrawn after having received a non-authorized reduced dose. Since thrombotic microangiopathy (TMA) occurred in two patients in the second step at dose level 2, the DSMB recommended to include exclusively non-nephrectomized patients in the third step to limit the risk of an induced TMA. A confirmatory cohort of six patients received treatments at DL1. Patients received the treatment during a median (range) duration of 6 (1.9–52.4) weeks. Treatment discontinuation was decided because of progression for 12 patients, adverse events for 12 other patients and investigator’s decision for one. The main reasons for discontinuation before the 24-week tumor assessment were disease progression (*n* = 7) or toxicity (*n* = 11). One patient with mRCC discontinued treatment since the resection of a single residual pancreatic metastasis was decided. Treatment-related adverse events led to pazopanib dose modifications in 11 of the 25 patients (eight dose interruptions and three dose reductions). Bevacizumab administration was delayed because of toxic effects in six patients, five at DL1 and one at DL2.

### Safety and MTD determination

The dose escalation and DLT are listed in Table 2. No DLT was observed within the initial cohort (DL1) of nine patients. Ten patients were enrolled at DL2 i.e. 600 mg pazopanib combined with 7.5 mg/kg bevacizumab, five DLT were observed. Two patients experienced a grade 3 hepatic cytolysis with ALT/AST elevation (ALT >6xULN and AST >3xULN; ALT >9xULN and AST >7xULN, respectively) associated with hyperbilirubinemia (total bilirubin >1.7xULN and >1.2xULN, respectively). A pulmonary embolism occurred in one patient. Two patients developed clinical features consistent with a microangiopathic hemolytic anemia (MAHA) syndrome with proteinuria, hemolytic anemia, low haptoglobin, thrombocytopenia, and serum creatinine increase, 4 weeks after pazopanib initiation and 2 weeks after the first bevacizumab infusion. To note, one of these patients was previously nephrectomized for his RCC and had a creatinine level above normal at baseline (122 μmol/L for a normal range upper value of 110 μmol/L); both patients had a history of hypertension.

**Table 2:** DLTs according to dose levels

Dose level	Number of Assessable patients	Bevacizumab (Q2W)	Pazopanib (Q.D)	Number of DLT
DL1	9	7.5 mg/kg	400 mg	No DLT
DL1 Confirmatory Cohort	6			3 DLT:- 2 grade 3 MAHA^b^
				- 1 grade 3 ALT/AST
DL2	10^a^	7.5 mg/kg	600 mg	5 DLT:- 2 grade 3 ALT/AST^c^
				- 1 grade 3 pulmonary embolism^c^
				- 2 grade 3 MAHA^c^

^a^1 patient dropped out for non-authorized dose reduction at week 5

^b^MAHA: Microangiopathic & hemolytic anemia

^c^occurred in one nephrectomized patient

In the six non-nephrectomized patients enrolled in the confirmatory cohort at DL1, one patient with metastatic melanoma was not assessable for MTD of the combination because of an early grade 3 thrombocytopenia during the first fortnight of pazopanib administration, and before any bevacizumab infusion. Grade 3 ALT/AST elevations were observed in one patient. Two patients developed a MAHA syndrome (grade 3) with proteinuria, hematuria, renal impairment, and thrombocytopenia; a renal biopsy confirmed the diagnosis of thrombotic microangiopathy in both cases. One occurred 4 weeks after pazopanib initiation and 2 weeks after the first bevacizumab injection, the other 6 weeks after pazopanib initiation and 1 week after the second bevacizumab injection. Patients had no history of hypertension and a normal renal function at baseline.

Adverse events (any grade) are shown in Table 3. The most frequently reported adverse events included fatigue (52%), hypertension (48%), anorexia (44%) and nausea (44%). The most frequently reported grade 3 and 4 adverse events included TMA (16%), thrombocytopenia (12%), abdominal pain (8%), thoracic musculoskeletal pain (8%), hypertension (8%) and proteinuria (8%) (Table 4). The occurrence of grade 3–4 events was equally represented in the first (*n* = 16) and in the second dose level (*n* = 14).

**Table 3:** Adverse events (all grades, occurring in >10% of patients). Total number of patients *N* = 25

	Grade					
	1	2	3	4	All	
	N	N	N	N	N	%
Fatigue	6	6	1	0	13	52.0
Hypertension	4	6	2	0	12	48.0
Anorexia	8	3	0	0	11	44.0
Nausea	11	0	0	0	11	44.0
Asthenia	6	4	0	0	10	40.0
Diarrhea	8	1	1	0	10	40.0
Dysphonia	10	0	0	0	10	40.0
Vomiting	7	2	1	0	10	40.0
Thrombocytopenia	3	2	3	0	8	32.0
Headache	6	0	0	0	6	24.0
Abdominal pain	2	2	2	0	6	24.0
Dysgeusia	4	2	0	0	6	24.0
Myalgia	5	1	0	0	6	24.0
Neck pain	4	1	0	0	5	20.0
Hypothyroidism	3	2	0	0	5	20.0
Proteinuria	0	3	2	0	5	20.0
Dry skin	4	1	0	0	5	20.0
Arthralgia	3	1	0	0	4	16.0
Elevated bilirubin	0	4	0	0	4	16.0
Muscular contractures	4	0	0	0	4	16.0
Hair modified color	4	0	0	0	4	16.0
Epistaxis	4	0	0	0	4	16.0
Hemorrhoids	3	1	0	0	4	16.0
Microangiopathy	0	0	4	0	4	16.0
Paresthesia	4	0	0	0	4	16.0
Stomatitis	2	2	0	0	4	16.0
Hand & foot syndrome	3	0	1	0	4	16.0
Mucositis	3	1	0	0	4	16.0
Elevated AST	2	1	0	0	3	12.0
Back pain	2	1	0	0	3	12.0
Musculoskeletal pain	2	1	0	0	3	12.0
Thoracic musculoskeletal pain	0	1	2	0	3	12.0
Dyspnea	3	0	0	0	3	12.0
Urinary tract infection	0	3	0	0	3	12.0
Neutropenia	0	3	0	0	3	12.0

**Table 4:** Grade 3/4 adverse events according to dose level and nephrectomy

	Dose level 1				Dose level 2				All *N* = 25	
	Nephrec-tomized *N* = 4	Non nephrec-tomized *N* = 11	All *N* = 15		Nephrec-tomized *N* = 3	Non nephrec-tomized *N* = 7	All *N* = 10			
	N	N	N	%	N	N	N	%	N	%
Thrombotic microangiopathy (TMA)	0	2	2	13.3	1	1	2	20.0	4	16.0
Thrombocytopenia	0	2	2	13.3	0	1	1	10.0	3	12.0
Elevated ALT and/or AST	0	1	1	6.7	1	1	2	20.0	3	12.0
Abdominal pain	0	1	1	6.7	0	1	1	10.0	2	8.0
Thoracic musculoskeletal pain	0	2	2	13.3	0	0	0	0.0	2	8.0
Hypertension	0	0	0	0.0	1	1	2	20.0	2	8.0
Proteinuria	1	0	1	6.7	0	1	1	10.0	2	8.0
Perianal abscess	1	0	1	6.7	0	0	0	0.0	1	4.0
Seizure	0	1	1	6.7	0	0	0	0.0	1	4.0
Diarrhea	0	0	0	0.0	0	1	1	10.0	1	4.0
Confusion^a^	0	1	1	6.7	0	0	0	0.0	1	4.0
Fatigue	0	1	1	6.7	0	0	0	0.0	1	4.0
Hypernatremia	0	0	0	0.0	1	0	1	10.0	1	4.0
Post-surgery bleeding	0	0	0	0.0	1	0	1	10.0	1	4.0
Lipase increase	0	0	0	0.0	1	0	1	10.0	1	4.0
Pyelonephritis	0	1	1	6.7	0	0	0	0.0	1	4.0
Hand & foot syndrome	1	0	1	6.7	0	0	0	0.0	1	4.0
Venous thrombosis	0	0	0	0.0	1	0	1	10.0	1	4.0
Vomiting	0	1	1	6.7	0	0	0	0.0	1	4.0

^a^Grade 4 adverse event

The DL1 (400 mg pazopanib-7.5 mg/kg bevacizumab) was defined as the MTD since no DLT was observed in the nine first patients treated at DL1 but three DLTs occurred in the six additional patients.

### Pharmacokinetics

The mean (range) plasma concentration (AUC) with 400 and 600 mg pazopanib administration were respectively 283 (139–427) (*n* = 15), and 494 (227–761) μg.h/mL (*n* = 10) at Day 1, and 738 (487–989) and 1071 (678–1464) μg.h/mL at Day 15, with 37% of inter-individual variability in apparent clearance. These values were significantly higher than those previously described in the initial phase 1 trial with pazopanib as monotherapy [37]. However, they were not influenced by bevacizumab infusion since pazopanib trough plasma concentrations were not significantly higher 24 h after bevacizumab infusion than before infusion (at D15). The detailed results were previously published [36].

### Efficacy

**Fig. 2:**
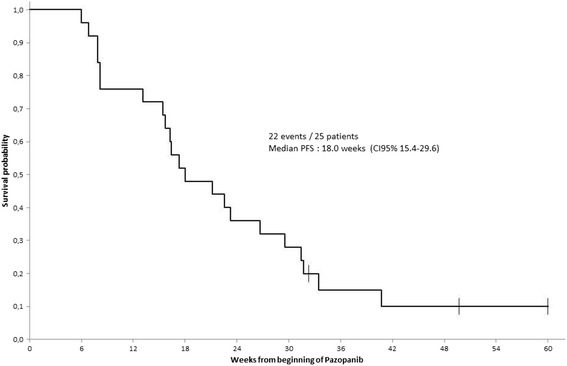
Progression-free Survival

As of the cutoff data for data analysis (Oct 7th, 2013), the median (range) follow-up was 11.4 months (1.8–25.8). Twenty-two patients were evaluable for response to treatment. To note, two patients stopped prematurely the experimental treatment for toxicity after a treatment period of 4 and 5 weeks respectively, and one patient never received bevacizumab. The best overall response observed was partial response (PR) in five (22.7%) patients (three at DL1 and two at DL2; responses occurring in mRCC, lung cancer, cervix cancer (*n* = 2), and seminoma patients), stable disease (SD) in 11 (50%) patients (eight at DL1 and three at DL2; five patients with mRCC and six with other tumors), and progressive disease (PD) in six (27.3%) patients (three at each DL; six patients with other tumors than mRCC). The 24-week progression-free rate was 33.3% (95% CI, 15.63–55.32%) in the whole study cohort, with a median PFS of 18 (95% CI, 15–30) weeks (Fig. 2). No difference in PFS was observed in patients at DL1 and those at DL2 (data not shown). Median PFS were 23.3 weeks (95% CI, 15.7–31.4) and 17.1 weeks (95% CI, 8.1–26.7) in patients with mRCC and other tumor types, respectively.

## Discussion

The combination of the VEGF-directed monoclonal antibody bevacizumab with a tyrosine kinase inhibitor directed against VEGF receptors has already been investigated [28]. These combination regimens were difficult to manage since increased toxicities have been described, and some of them were even considered as not feasible despite promising efficacy. The more recently registered TKI pazopanib, appeared to induce fewer side effects than other previous VEGFR TKIs [8, 38]. Most patients with mRCC also preferred pazopanib to sunitinib [39]. The combination of pazopanib and bevacizumab appeared feasible and a promising efficacy was expected. This trial is the first to report this combination in patients with different solid tumor including mRCC patients.

The MTD was 400 mg pazopanib and 7.5 mg bevacizumab, defined as the initial DL combination to investigate in this trial. These doses are equivalent to one half of pazopanib and three-quarters of bevacizumab doses recommended when administrated as monotherapy. AST or ALT increases to up to a grade 3 toxicity level were considered as a DLT in three patients. This severe hepatotoxicity was already described in trials investigating pazopanib as monotherapy [8, 38, 40, 41]. This VEGFR TKI appears to commonly induce some hepato-toxicity. However, the rate of hepatic toxicity was notably higher in the large pazopanib phase III trial (*N* = 557), than that observed with the combined treatment [38]. Hepatic toxicity might therefore not be linked to the combination under investigation. On the opposite, the occurrence of a microangiopathic hemolytic anemia (MAHA), initially reported with bevacizumab but also observed with sunitinib, might be favored by the combination of both agents targeting the VEGF pathway [42–44]. Four patients with MAHA syndrome were observed in our series; two occurred at DL2 in patients with a history of hypertension, one of them previously underwent a nephrectomy and had an increased creatinine level before receiving the treatment. MAHA syndromes were also observed in two patients within the non-nephrectomized additional cohort of patients treated at DL1. Both occurred in patients with no history of hypertension and with previously normal renal function. Our results demonstrate that this vasculo-renal impairment can be induced in patients without any vascular or renal pre-existing risk. Thrombotic microangiopathy (TMA), as the initial phenomenon in the development of a MAHA syndrome, has been reported by several authors investigating the combination of sunitinib and bevacizumab [25, 28, 45]. On the contrary, another registered TKI directed against VEGFR, sorafenib, combined with bevacizumab did not mention any occurrence of MAHA syndrome nor TMA [14, 16, 19]. Pazopanib combined with bevacizumab might damage the renal nephron, with a rapid onset of a microangiopathy closed to that reported in the combination of bevacizumab with sunitinib [28]. In addition, our series confirmed that this microangiopathic effect does not only occur in patients suffering from renal tumors.

To note, no significant change in pazopanib PK was noticed following bevacizumab administration, especially in patients who experienced severe adverse events [36]. Moreover, the mean daily AUC at Day 15 (i.e., 1071 ± 398 μg.h/mL) at the maximum dose tested (600 mg) in combination with bevacizumab was higher in this trial than that determined in the first-in-man phase 1 study with 800 mg pazopanib once-daily administration as monotherapy (i.e. 743 ± 76 μg.h/mL, *n* = 8) [37]. This could be related to a different patient selection and may explain the poor tolerance we observed, imposing 400 mg pazopanib as the maximum tolerated dose in the combination with bevacizumab.

Beside these dose limiting toxicities, the type and frequency of other adverse events were similar to those observed with pazopanib as monotherapy in large randomized trial [38].

Even if 22% patients achieved a partial response, the response rate in the six mRCC patients, with one responding patient only, was not promising despite the administration of this combination in first-line setting. Interestingly, several objective responses were observed in other heavily pretreated tumors. If bevacizumab is already part of the treatment strategy used in cervix and lung carcinomas [3, 46], a significant tumor reduction in a patient with a seminoma was more surprising.

## Conclusions

The combination of pazopanib and bevacizumab displays significant toxicity. This combination required the use of reduced doses compared to their respective monotherapy administration. A microangiopathy was observed in some patients without any specific pre-existing vascular or renal conditions. The safety issues, together with a rather disappointing efficacy rate, preclude the further development of this combination.

## Abbreviations

AE: Adverse event
ALT: Alanine aminotransferase
APPT: Activated partial thromboplastin time
AST: Aspartate aminotransferase
CTCAE: National Cancer Institute Common Terminology Criteria for Adverse Events
DL: Dose levels
DLT: Dose limiting toxicities
DSMB: Independent Data and Safety Monitoring Committee
ECOG-PS: Eastern Cooperative Oncology Group performance status
MAHA: Microangiopathic hemolytic anemia
mRCC: Metastatic renal cell carcinoma
MTD: Maximum tolerated dose
PDGFR: Platelet-derived-growth-factor receptor
PK: Pharmacokinetics
PT: Prothrombine time
TKI: Tyrosine kinase inhibitors
TMA: Thrombotic microangiopathy
ULN: Upper limit of normal [ULN]
VEGF: Vascular endothelial growth factor
VEGFR: VEGF receptors
